# Supplementary figures and images for: Comparative analysis of DNA extraction and PCR product purification methods for cervicovaginal microbiome analysis using cpn60 microbial profiling
Source: PLoS One. 2022 Jan 13;17(1):e0262355. doi: 10.1371/journal.pone.0262355 (PMC8758110; doi:10.1371/journal.pone.0262355)

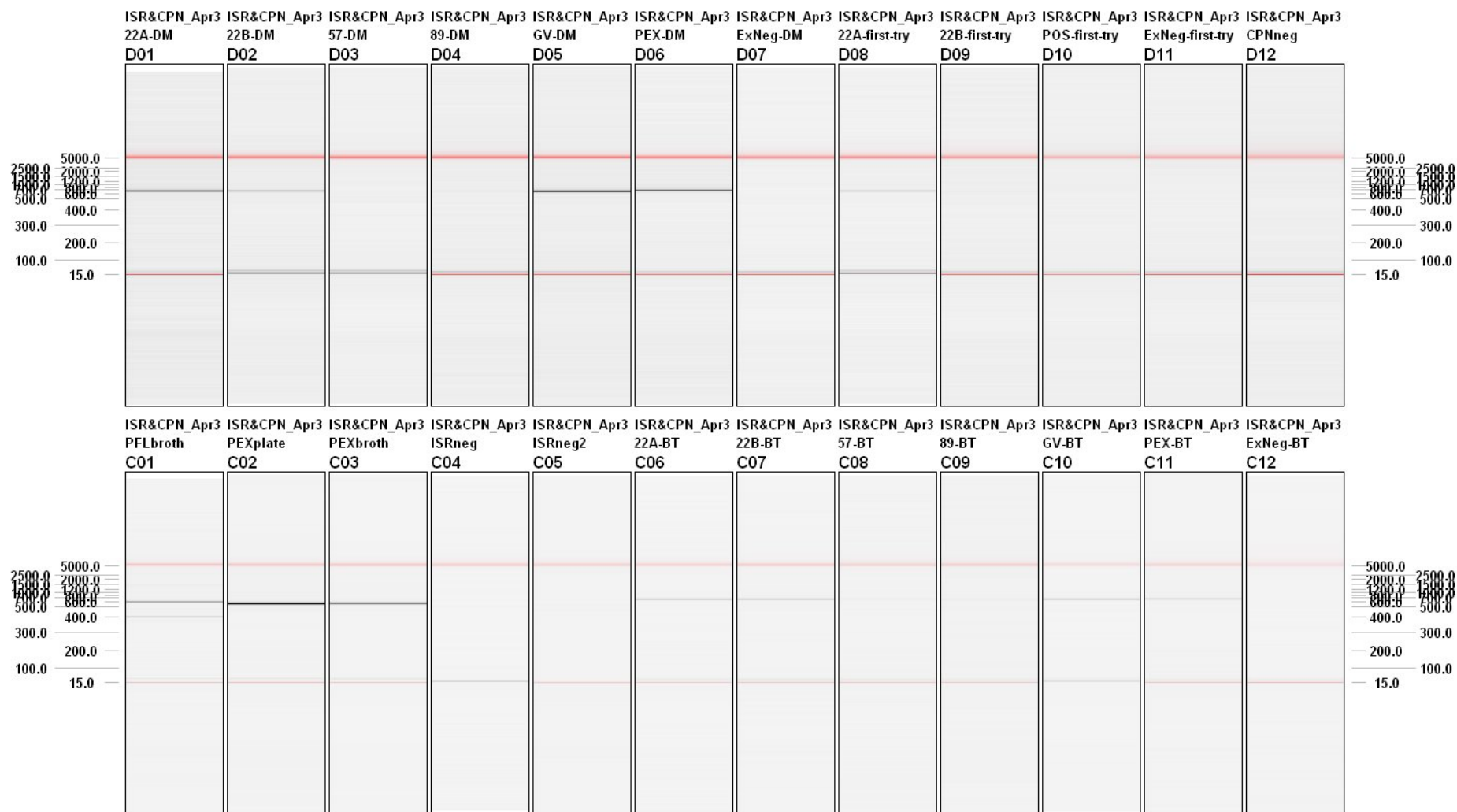

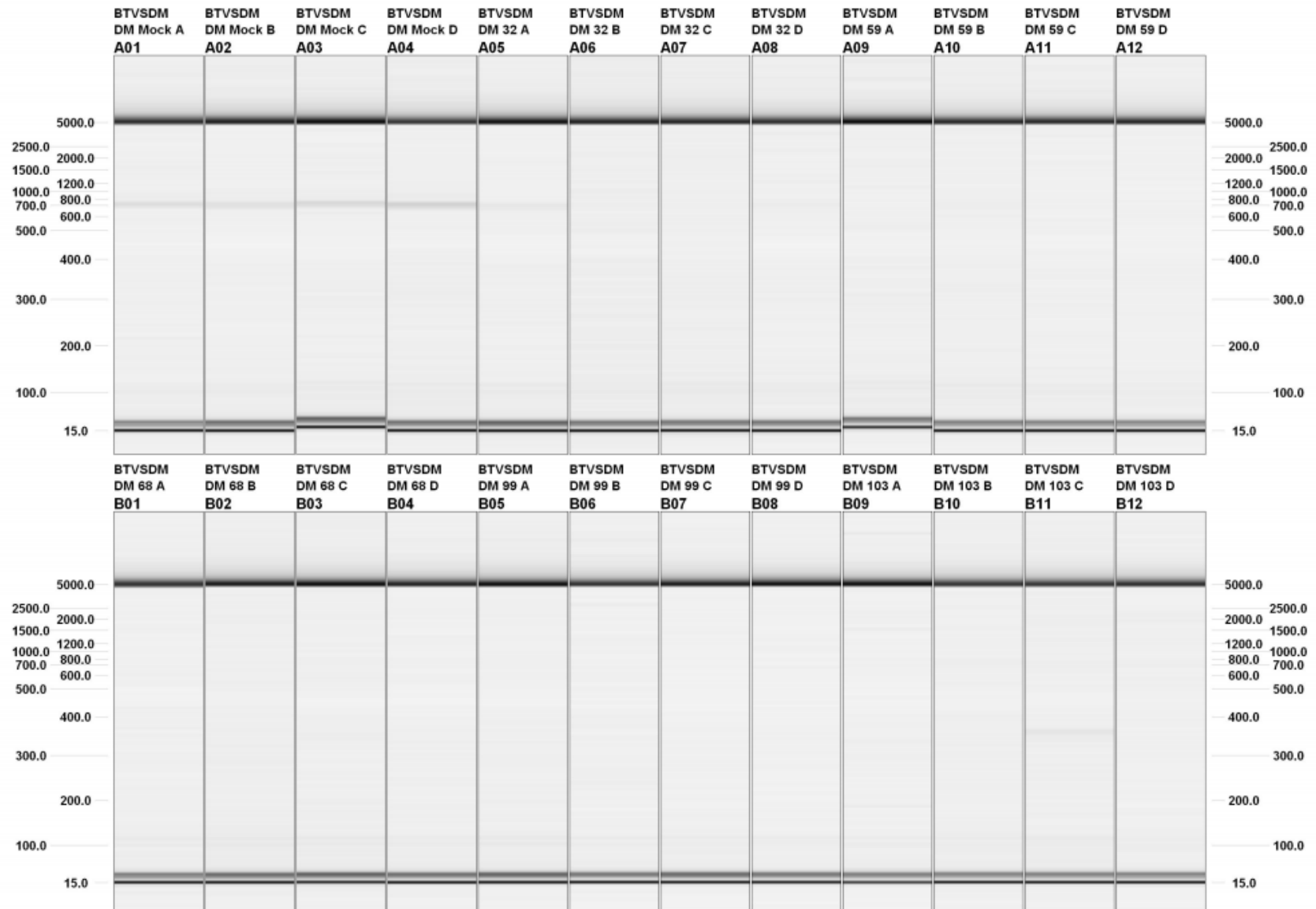

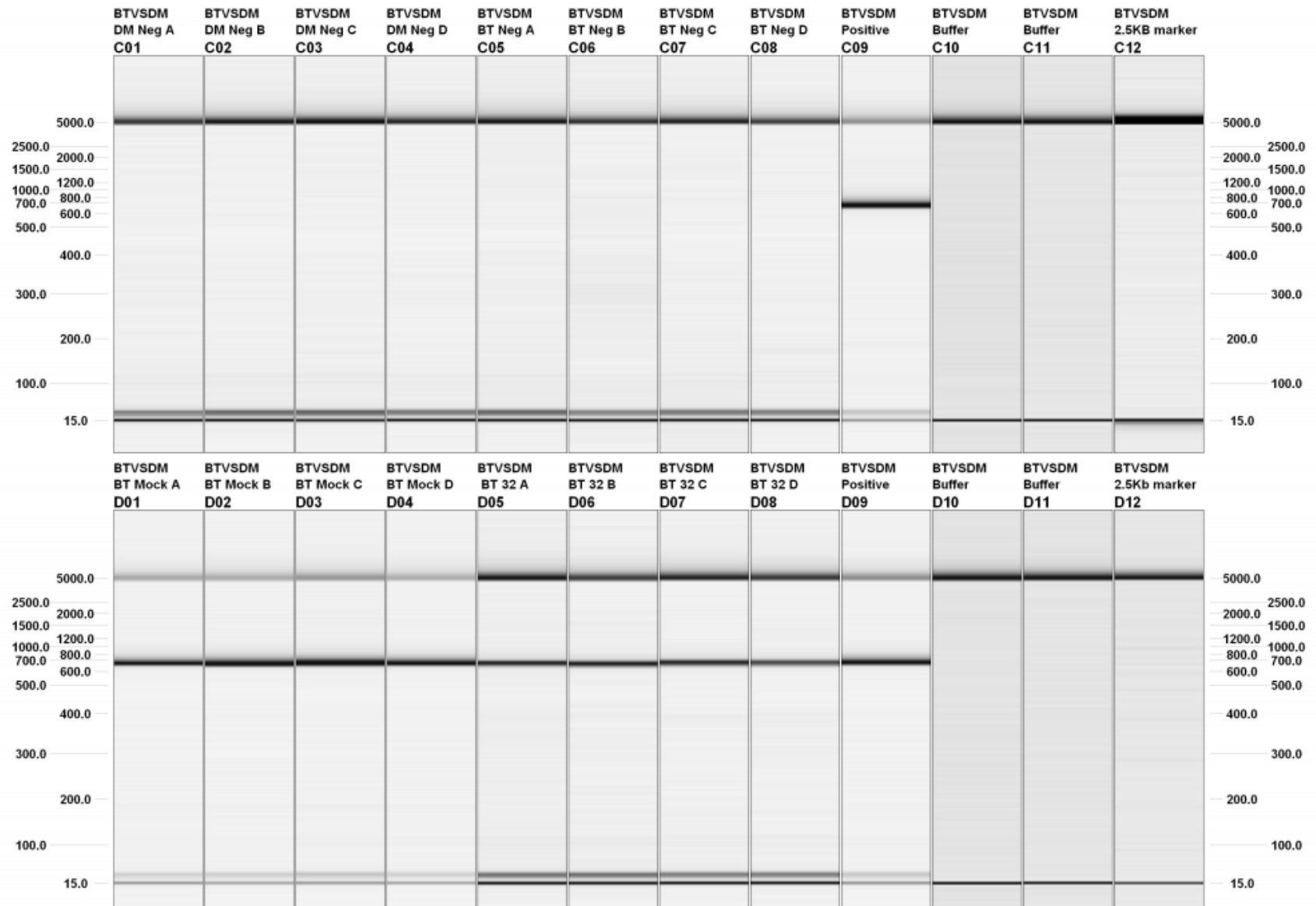

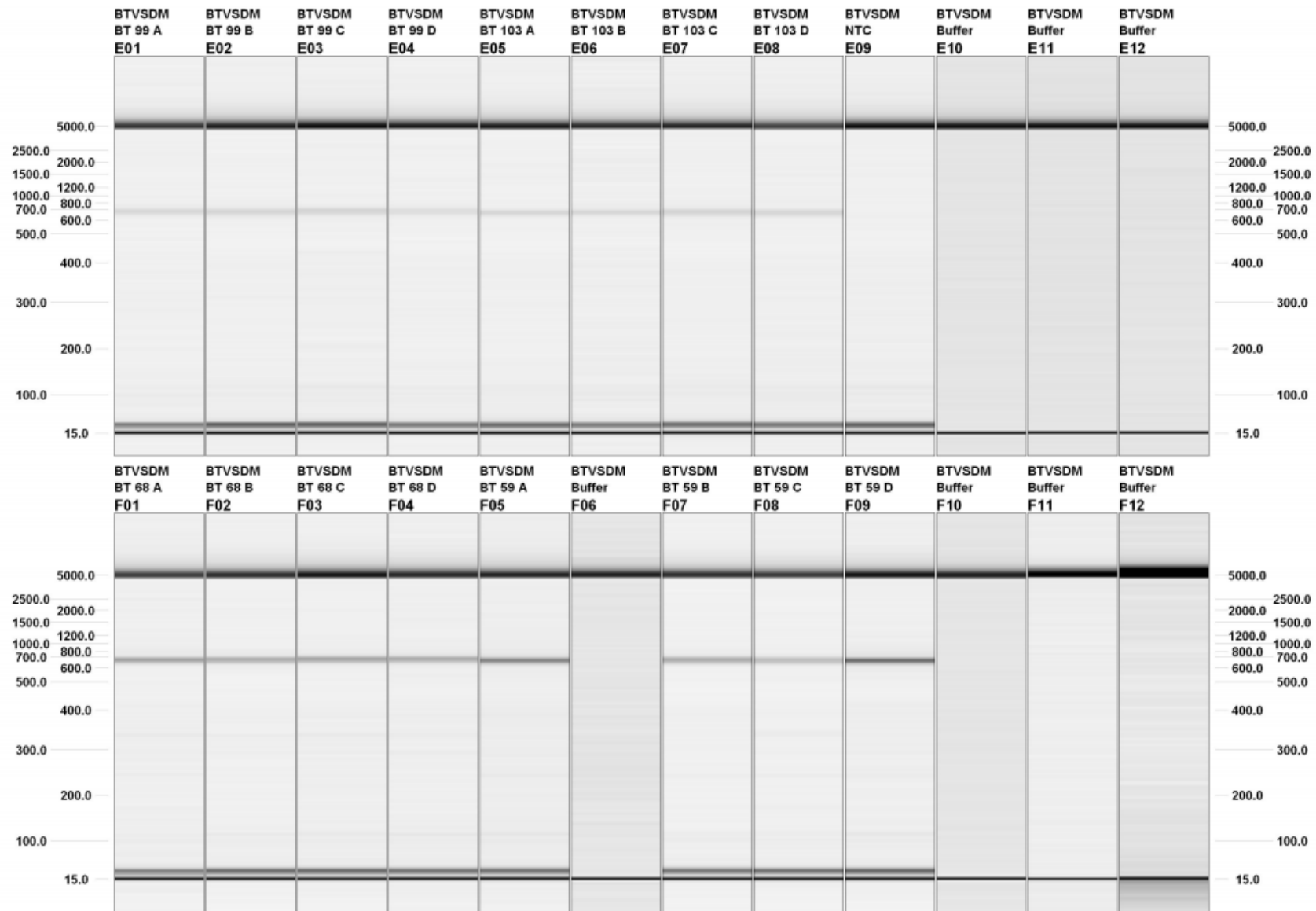

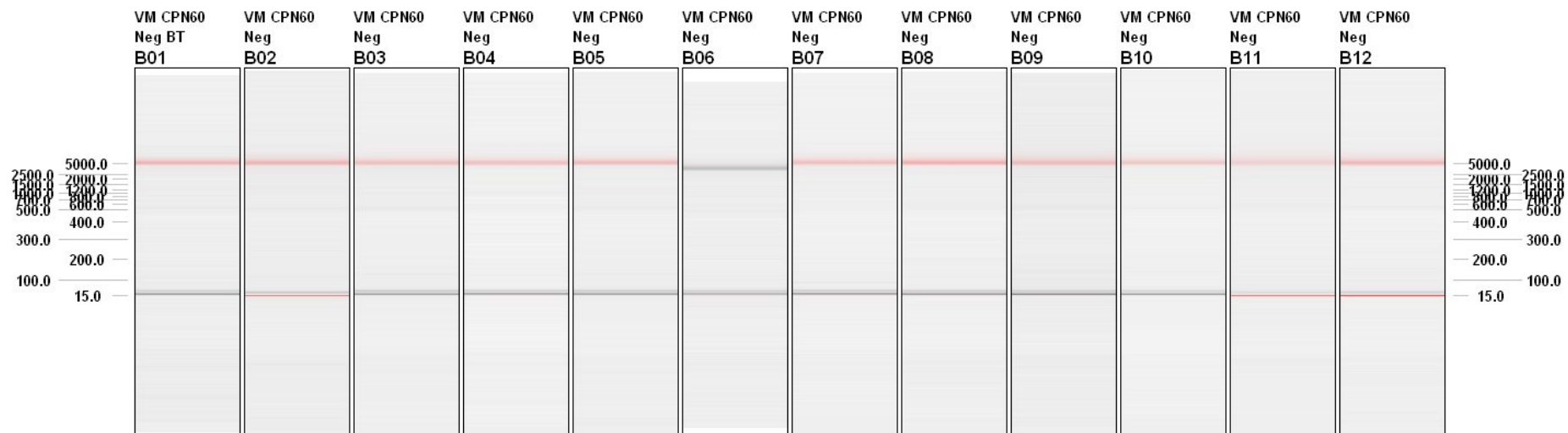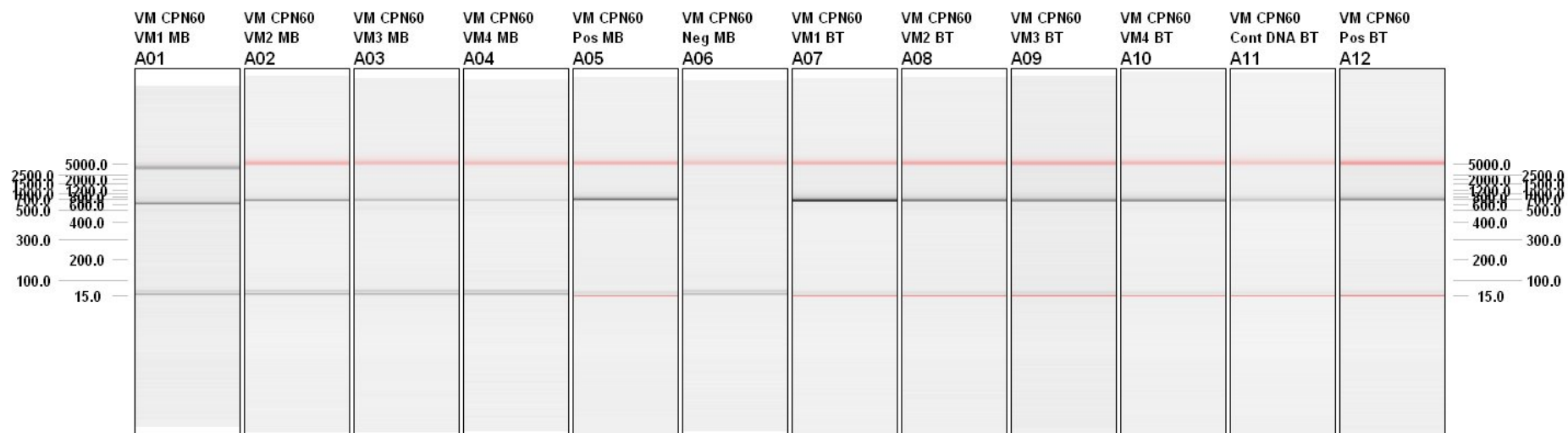

Supplement: S1 Fig — DM or MB refer to QIAamp DNA Microbiome kit, BT, modified DNeasy Blood and Tissue kit protocol. Numbers refer to VZV participant ID unless otherwise specified. Note that some samples were amplified alongside our study samples using cpn60 for another unrelated study and are also included in these raw images (for ex: cont DNA BT). (PDF) [file pone.0262355.s002.pdf]
