# Supplementary material for: Comparative analysis of DNA extraction and PCR product purification methods for cervicovaginal microbiome analysis using cpn60 microbial profiling
Source: PLoS One. 2022 Jan 13;17(1):e0262355. doi: 10.1371/journal.pone.0262355 (PMC8758110; doi:10.1371/journal.pone.0262355)
Supplement: Attachment — Submitted filename: Response to reviewers.pdf [file pone.0262355.s005.pdf]

University of Manitoba | Rady Faculty of Health Sciences

Department of Internal Medicine,
Section of Infectious Diseases Room
501, 745 Bannatyne Ave Winnipeg MB,
Canada R3E 0J9 T: 204 977 5681

November 25, 2021

Prof Guangming Zhong MD PhD
Academic Editor
Plos One

Dear Dr. Guangming Zhong.

Re: Revisions to *Shvartsman E. et al*. entitled:
Comparative analysis of DNA extraction and PCR product purification methods for cervicovaginal microbiome analysis using cpn60 microbial profiling

On behalf of my co-authors, I would like to thank you and the reviewers for their useful comments on our manuscript. We appreciate the feedback and have made every effort to incorporate the suggestions in the manuscript. Please find below the description of the changes made to the manuscript in response to the reviewers' comments and suggestions. The reviewers' comments are indicated in bold print and our responses are italicized. As promised, the study sequencing data has been deposited in the NCBI sequence read archive (SRA) and can be accessed through the following BioProject accession: PRJNA783648. The reference list has been edited to reflect the PLoS One formatting requirements.

We trust that we have adequately responded to the concerns of the reviewers and look forward to your response to this revised manuscript.

Best Regards,

Kelly S. MacDonald MD FRCPC
H.E. Sellers Research Chair
Infectious Diseases Consultant
Professor of Medicine & Medical Microbiology
University of Manitoba

Response to Reviewers

Response to reviewer #1:

Reviewer #1: This manuscript is written very well. The design is studied well. I do think comparing two different sets of experimental methods confounds the results a bit. Perhaps it would have been better to compare microbial yield using the two different DNA extraction kits and then only using either the column-based method or the gel-based method, instead of also comparing that second element. Inclusion of the mock community standard was a good thought.

We thank the reviewer for their kind words and agree that the added method comparison did add an additional layer of complexity with respect to the analysis. This has been addressed within the manuscript by addition of replicates for both extraction and PCR-clean up methods, and has been addressed during statistical analysis, where paired comparisons were performed where applicable.

Response to reviewer #2:

Reviewer #2: 1) I have to say the manuscript is very unclear. I cannot even tell how many samples were involved and how many replicates were done. Across figures, why the samples are changed? There should be a good catalogue on what samples underwent what experiments.

We appreciate the reviewer's comments and think the inclusion of a sample catalogue and additional clarifications within the text have made the paper better organized. A sample catalogue has been included as a supplementary table to address questions regarding replicate number and status for the biological samples and mock standards (S1 Table) per extraction method and PCR-purification method. Footnotes have been added in the table where applicable.

We acknowledge that there was variation in the number of replicates per sample. This was due to different persons performing the extractions and has been addressed where applicable in the figures and in the statistical analysis where error bars have been included where applicable.

Some samples were indeed omitted from the PCR-product clean-up and the final sequencing round due to failure to amplify with one or both kits – this has been addressed in S1 Table, shown in S1 Fig, and stated within the text on lines 195-196 in the methods section and on lines 261-264 in the results section. Since the purpose of the study was to compare two extraction methods, failure to amplify with one kit resulted in omission of the sample from any downstream processes (ex: PCR clean-up, and sequencing).

2) Sequencing depth can be related with sequencing library preparation. How can it be accounted for before comparing the sequencing results, which is related with sequencing depth, between extraction and purification methods?

We agree with the reviewer that sequencing depth (or the total number of read output per sample) is indeed related to library preparation. In our study, library preparation was consistent

across all sequenced samples (same cpn60 PCR protocol was used, same index PCR, post-index PCR, etc). This has been further clarified on lines 213-214 in the text.
